# Elevated levels of lipoprotein(a) and low-density lipoprotein cholesterol in familial hypercholesterolemia patients: is dual primary prevention already in sight?

**DOI:** 10.3389/fcvm.2025.1624049

**Published:** 2025-07-01

**Authors:** Alpo Vuorio, Frederick J. Raal, Petri T. Kovanen

**Affiliations:** ^1^ Mehiläinen Airport Health Centre, Vantaa, Finland; ^2^ Department of Forensic Medicine, University of Helsinki, Helsinki, Finland; ^3^ Faculty of Health Sciences, University of Witwatersrand, Johannesburg, South Africa; ^4^ Cardiovascular Research Laboratory, Wihuri Research Institute, Helsinki, Finland

**Keywords:** lipoprotein(a), plaques, atherosclerotic cardiovascular disease, low-density lipoprotein cholesterol, familial hypercholesterolemia

Heterozygous familial hypercholesterolemia (HeFH) is a genetic disorder that is characterized by a lifelong elevation of low-density lipoprotein cholesterol (LDL-C) due to impaired clearance by dysfunctional LDL receptors (1). About 30% of HeFH subjects also have elevated levels of another genetically determined atherogenic lipoprotein, namely lipoprotein(a) [(Lp(a)] (1). With a worldwide prevalence of HeFH of approximately 1 in 300 (2, 3), it can be estimated that there are over 10 million HeFH patients with an elevated Lp(a) level over 50 mg/dl (>125 nmol/L). As both elevated LDL-C and Lp(a) are associated with an increased risk for premature atherosclerotic cardiovascular disease (ASCVD), in those HeFH patients with both conditions, the risk of premature ASCVD is likely to be even greater (4). Importantly, Bhatia and coworkers (2025) have shown that in statin-treated patients, when compared with an Lp(a) level of 5 mg/dl, higher levels of Lp(a) are log-linearly associated with increasing ASCVD risk.

Current guidelines recommend that statin therapy be initiated in childhood in subjects diagnosed with HeFH (5). However, no clear recommendations exist for treating elevated Lp(a) in subjects with HeFH. Currently, an oral small molecule Lp(a) inhibitor and several Lp(a) lowering antisense oligonucleotide and siRNA formulations are in clinical trials (6–11). The current trials involve only adult subjects, and the question has been raised whether Lp(a)-lowering drugs should also be considered and tested in young HeFH patients (12). Below, we will critically review the evidence that elevated Lp(a) may accelerate the development of atherosclerosis in young HeFH individuals, and if it does, then this would support pharmacological primary prevention for elevated Lp(a) in young HeFH patients.

In a recent Norwegian study, Lp(a) levels were measured in 438 children with genotypically confirmed HeFH, and approximately 24% of the girls and 17% of the boys in this cohort had elevated Lp(a) levels above 50 mg/dl (approximately 125 nmol/L), i.e., a level considered to be significantly atherogenic (13). In children with HeFH of either sex, increased Lp(a) concentrations are therefore more prevalent than in the general population. In addition, in a large cross-sectional study of 1,960 patients with HeFH and their 957 non-HeFH relatives, higher Lp(a) levels were found in those with HeFH, particularly in those with pre-existing ASCVD, compared to their non-HeFH relatives (14) suggesting that elevated Lp(a) levels predispose to a heightened risk of ASCVD in patients with HeFH.

Regarding the pathophysiological importance of an elevated Lp(a) level, impaired endothelium-dependent dilation has been observed in HeFH children as young as seven years old (15). Furthermore, in the study by Charakida et al. (16), inflammatory and hemostatic abnormalities that associated with vascular dysfunction were present in HeFH children with elevated Lp(a) levels but not in those HeFH children without. An additional interesting piece of information comes from the study by Hegele and coworkers (17), who studied stress thallium scans in HeFH children with a family history of premature ASCVD. They found that in HeFH children aged 9–23 years, Lp(a) levels tended to be higher in those in whom the stress thallium heart scan revealed a reduced coronary blood flow. Moreover, in a recent 20-year follow-up study of 200 HeFH children aged 8–18 years who participated in a statin trial in which the carotid intima-media thickness was serially measured as an indicator of subclinical atherosclerosis it appeared that a high Lp(a) level contributed significantly to the progression of carotid intima-media thickness [β adjusted 0·0073 mm per 50 nmol/L increase in lipoprotein(a); 95% CI (0·0013–0·0132); *p* = 0·017] (18). The authors of this study concluded that an elevated level of Lp(a) is an independent risk factor for early atherosclerosis and that, therefore, it is essential to measure Lp(a) in young patients with HeFH. Despite this interesting finding which strongly supports Lp(a) as a risk factor for early atherosclerosis in HeFH patients, a very recent study of 143 young adults with HeFH (age 31.8 ± 3.2 years) failed to find an association with carotid arterial stiffness and Lp(a) levels (12). However, arterial stiffness may not be a good measure of early atherosclerosis in young adults, and other surrogate markers of early signs of atherosclerosis, such as plaque formation, may be more suitable to evaluate the Lp(a)-mediated contribution to atherosclerosis in young FH patients.

Despite lipid-lowering treatment (LLT), exposure to even moderately increased LDL-C levels from birth in HeFH patients results in premature development of ASCVD, particularly coronary atherosclerosis. This was eloquently shown in a study in which 90 HeFH patients (mean age 41 ± 3 years) having LLT initiated before or after age 25 were compared to an age- and sex-matched unaffected control group (19). FH patients had a higher cumulative LDL-C exposure (181 ± 54 vs. 105 ± 33 mmol/L ∗ years) and higher prevalence of coronary plaque compared with controls [46 (51%) vs. 10 (22%), OR 3.66 (95% CI 1.62–8.27)]. Every 75 mmol/L ∗ years, cumulative exposure to LDL-C was associated with a doubling in percent atheroma. Early treated patients had a modestly lower cumulative LDL-C exposure than did late-treated FH patients (167 ± 41 vs. 194 ± 61 mmol/L ∗ years; *P* = 0.045), without significant difference in coronary atherosclerosis. This study emphasizes the need for early initiation of intensive lipid-lowering treatment in subjects with HeFH.

In a recent study by Shishikura et al. (20) analyzed 439 patients with coronary artery disease (CAD) using near-infrared spectroscopy (NIRS) imaging, which enables the quantitative evaluation of lipidic-plaque materials. NIRS-derived maximum 4 mm lipid-core burden index (MaxLCBI_4 mm_) ≥ 400 is associated with an increased risk of cardiovascular events (19). In this study the coexistence of LDL-C < 70 mg/dl and Lp(a) < 50 mg/dl showed an approximately 70% lower risk (adjusted odds ratio: 0.30; 95% confidence interval: 0.13–0.68) of MaxLCBI_4 mm_ ≥ 400 when compared with the reference group [LDL-C ≥ 70 mg/dl and Lp(a) ≥ 50 mg/dl] (20). Thus, the findings of this study strongly support the idea of treating Lp(a) to stabilize atherosclerotic plaques.

The findings of a very recent study suggest that the optimal cut-off point for Lp(a) in HeFH patients needs to be below 10 mg/dl (approximately 25 nmol/L), i.e., a level which is significantly lower than that associated with increased ASCVD risk in the general population (above 30 mg/dl or 75 nmol/L) (21). Importantly, since the atherosclerosis-promoting effect of a high Lp(a) level, like that of a high LDL-C level, is present from birth, HeFH patients with elevations in both LDL-C and Lp(a) may present with their first cardiovascular event before the age of 35 years (22).

Worldwide, FH remains underdiagnosed and, even if diagnosed, undertreated or untreated, with most patients only having LLT aimed at lowering LDL-C commenced in adulthood. As discussed above, this is especially deleterious for those FH patients who, in addition to the high LDL-C levels, also have high Lp(a) levels and are, therefore, even at greater risk for an ASCVD event. However, treatment aimed at concurrently lowering Lp(a) is presently not considered. Notably, a recent German Lipoprotein Apheresis Registry follow-up study involving non-FH patients with an isolated elevated Lp(a) level showed that the patients benefited from a reduction of the Lp(a) level in that the number of ASCVD events was reduced (23). So, in FH patients whose coronary atherosclerotic plaques may be stenotic and show characteristics of advanced vulnerability already in the teens, specific Lp(a)-lowering drug therapy should be considered and should accompany an early and effective LDL-C-lowering strategy to prevent or at least delay, the onset of premature ASCVD.

It has already been demonstrated in adult non-HeFH patients that the addition of the PCSK9 inhibitor, alirocumab, reduces major acute cardiovascular events, including coronary heart disease death, non-fatal myocardial infarction, fatal/non-fatal ischemic stroke, unstable angina requiring hospitalization to a greater degree among those patients who had a high baseline Lp(a) level (24). Therefore, the availability of the PCSK-9 inhibitors seems to offer a unique opportunity for primary prevention in HeFH children with a double heritable risk (25). Including a PCSK9 inhibitor in the intensive treatment schedule necessary for these very high-risk HeFH children with both elevated LDL-C and Lp(a) levels should be considered. However, because of their additive lifelong cumulative burden, it remains to be proven whether an optimal early primary prevention of HeFH necessitates targeting both atherogenic lipoproteins (26) (Figure 1).

**Figure 1:**
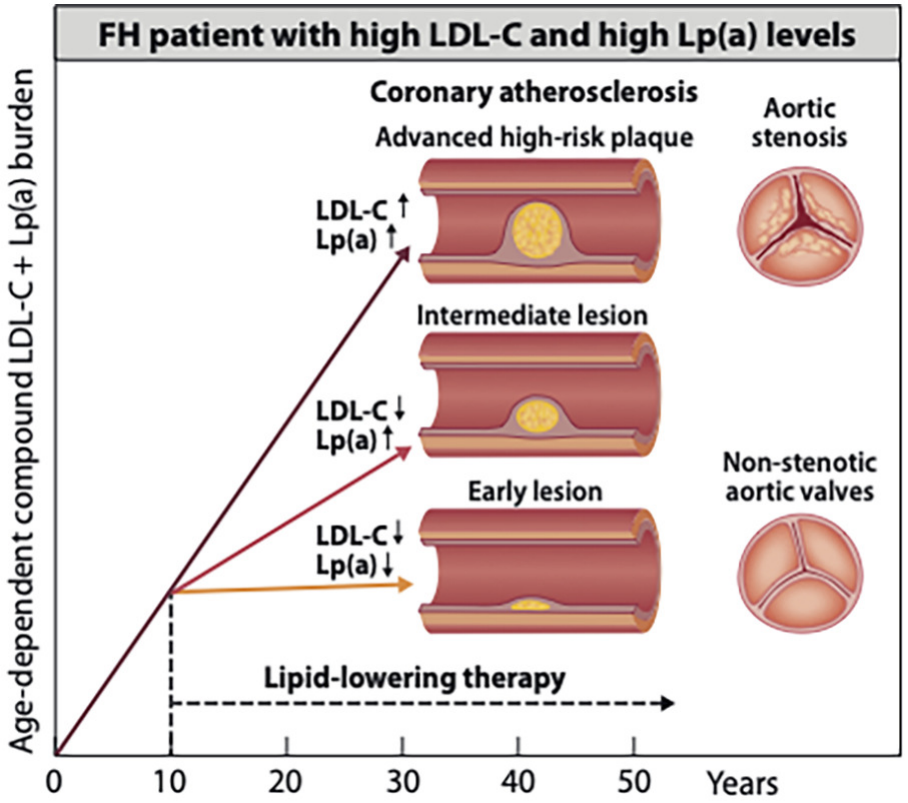
Potential beneficial effects of early initiation of pharmacotherapies to delay the onset of atherosclerosis in an HeFH patient with elevation of both LDL-C and Lp(a) potential beneficial effects of early started pharmacotherapies on the development of coronary atherosclerosis in an HeFH patient with elevation of both LDL-C and Lp(a) level. About one-third of patients with HeFH have since birth a genetically determined elevation of both low-density lipoprotein cholesterol (LDL-C) and lipoprotein (a) [(Lp(a)]. Such joint elevation of two atherogenic lipoproteins strongly accelerates the development of atheroscleroticcardiovascular disease (ASCVD) and the rates of ASCVD events. The figure shows illustratively that in an untreated HeFH patient as young as 40 years old, an advanced high-risk coronary plaque may develop. In contrast, when efficient LDL-C-lowering or combined LDL-C- and Lp(a)-lowering pharmacotherapy is already started in early childhood, only an intermediate or an early lesion may have developed by early middle age. On the right side of the image, severely stenotic and non-stenotic aortic valves are shown. Although aortic stenosis in HeFH without elevated Lp(a) is rare, a joint elevation of Lp(a) is likely to increase the risk for aortic stenosis (27). Accordingly, an early start of lowering LDL-C and, particularly, of Lp(a) is expected to reduce the risk of developing this disease condition.

## Summary

• Heterozygous familial hypercholesterolemia (HeFH) is characterized by a lifelong elevation of low-density lipoprotein cholesterol (LDL-C).
• About 30% of HeFH subjects also have elevated levels of another atherogenic lipoprotein, namely lipoprotein(a) [(Lp(a)].
• Currently, an oral small molecule Lp(a) inhibitor and several Lp(a) lowering antisense oligonucleotides and siRNA formulations are in clinical trials for the treatment of elevated Lp(a).
• Elevated Lp(a) impairs endothelium-dependent dilation, and this has been observed in HeFH children as young as seven years old.
• High Lp(a) level has been shown to contribute significantly progression of carotid intima-media thickness in HeFH children aged 9 to 23 years.
• The findings of a very recent study suggest that the optimal cut-off point for Lp(a) in HeFH patients needs to be below 10 mg/dL.
• In optimal early primary prevention of HeFH both elevated LDL-C and Lp(a) levels should be considered, because of their additive lifelong cumulative burdens.
